# Image quality assessment using deep learning in high b-value diffusion-weighted breast MRI

**DOI:** 10.1038/s41598-023-37342-3

**Published:** 2023-06-29

**Authors:** Lorenz A. Kapsner, Eva L. Balbach, Lukas Folle, Frederik B. Laun, Armin M. Nagel, Andrzej Liebert, Julius Emons, Sabine Ohlmeyer, Michael Uder, Evelyn Wenkel, Sebastian Bickelhaupt

**Affiliations:** 1 Institute of Radiology, Universitätsklinikum Erlangen, Friedrich-Alexander-University Erlangen-Nürnberg (FAU), Maximiliansplatz 3, 91054 Erlangen, Germany; 2 Medical Center for Information and Communication Technology, Universitätsklinikum Erlangen, Krankenhausstraße 12, 91054 Erlangen, Germany; 3 Pattern Recognition Lab, Friedrich-Alexander-University Erlangen-Nürnberg (FAU), Martensstraße 3, 91058 Erlangen, Germany; 4 Department of Obstetrics and Gynaecology, Universitätsklinikum Erlangen, Friedrich-Alexander-University Erlangen-Nürnberg (FAU), Universitätsstraße 21-23, 91054 Erlangen, Germany; 5 German Cancer Research Center (DKFZ), Im Neuenheimer Feld 280, 69120 Heidelberg, Germany

**Keywords:** Population screening, Breast cancer, Translational research

## Abstract

The objective of this IRB approved retrospective study was to apply deep learning to identify magnetic resonance imaging (MRI) artifacts on maximum intensity projections (MIP) of the breast, which were derived from diffusion weighted imaging (DWI) protocols. The dataset consisted of 1309 clinically indicated breast MRI examinations of 1158 individuals (median age [IQR]: 50 years [16.75 years]) acquired between March 2017 and June 2020, in which a DWI sequence with a high b-value equal to 1500 s/mm^2^ was acquired. From these, 2D MIP images were computed and the left and right breast were cropped out as regions of interest (ROI). The presence of MRI image artifacts on the ROIs was rated by three independent observers. Artifact prevalence in the dataset was 37% (961 out of 2618 images). A DenseNet was trained with a fivefold cross-validation to identify artifacts on these images. In an independent holdout test dataset (n = 350 images) artifacts were detected by the neural network with an area under the precision-recall curve of 0.921 and a positive predictive value of 0.981. Our results show that a deep learning algorithm is capable to identify MRI artifacts in breast DWI-derived MIPs, which could help to improve quality assurance approaches for DWI sequences of breast examinations in the future.

## Introduction

Mammography screening programs have successfully been implemented to reduce breast cancer-related mortality in females^1^. In breast imaging, magnetic resonance imaging (MRI) has mostly been used for screening in women with a hereditary breast cancer risk^2, 3^. MRI examinations of the female breast are routinely performed using a multiparametric approach. Herein, MRI protocols consist of anatomical, non-contrast enhanced sequences and dynamic contrast enhanced (DCE) sequences after the administration of gadolinium containing intravenous contrast agents^4^. More recently, complementary MRI techniques such as diffusion weighted imaging (DWI) have evolved demonstrating a clinical potential for breast cancer screening^5^. DWI sequences reflect the random Brownian motion of water molecules within the tissue. The diffusion process herein has been suggested to correlate to distinct microstructural features of the tissue, e.g., the cellularity or microstructural complexity. With this correlation, DWI is of special interest in oncologic imaging allowing to detect and characterize alterations of diffusion processes within breast tissue^6, 7^. Several studies have demonstrated the increased diagnostic accuracy of DWI in complementing the multiparametric MRI for the diagnosis of breast cancer^8^.

Recently, abbreviated breast MRI protocols have been evaluated to improve the applicability of breast MRI in high-throughput settings, such as screening examinations^9, 10^. Herein, mostly contrast enhanced protocols are considered. However, potential side effects of intravenous application of gadolinium containing contrast agents have been discussed in the last years^11–14^, leading to the suspension of some linear contrast agents in Europe^15^. With this, increasing interest has emerged in non-contrast enhanced imaging techniques, such as DWI. Initial studies suggested that abbreviated non-contrast enhanced DWI MRI protocols might provide diagnostic value, however, mostly not reaching the outstanding sensitivity of DCE MRI due to the technical challenges of DWI^16^. While DWI can be performed on most state-of-the-art MRI scanners, achieving a high diagnostic quality and respective quality consistency over time remains a technical challenge in clinical routine. DWI sequences are prone to image artifacts, which may be introduced, for example, by patient motion, insufficient fat saturation, image distortion, and blurring^17^. This currently impedes the diagnostic assessment and limits the potential of DWI in clinical routine.

The application of DWI in breast imaging is gaining interest and first approaches are already investigating the stand-alone-value of the technique. In this context, both quantitative and artificial intelligence (AI) augmented evaluation techniques are becoming more important, for which advanced quality assurance and artifact assessment technologies would be beneficial.

Similar to the application in abbreviated breast DCE-MRI protocols, maximum intensity projections (MIP) can also be computed from DWI sequences in order to reduce the radiologist’s initial reading time^16, 18, 19^. Since MIPs might accumulate (hyperintense appearing) artifacts from the single slices and thus impede the diagnostic assessment if used as an initial visualization approach for lesion detection, we here investigate the capability of a convolutional neural network (CNN) to detect artifacts occurring on high b-value DWI-derived MIPs in a large dataset as a preparatory groundwork for possible future application in abbreviated breast DWI-MRI protocols.

## Results

### Study cohort and demographics

A total of 1309 clinically indicated breast MRI examinations fulfilled the inclusion criteria, corresponding to a total of n = 1158 patients (median age at first acquisition: 50 years [IQR: 16.75 years]) that were included in the study. Demographic data and sample characteristics are shown in Table 1. 1020 individuals of the study sample received one, 125 individuals received two, and 13 individuals received a total of three MRI examinations within the study period. The training dataset included 1134 examinations of 984 patients (median age at first acquisition: 50 years [IQR: 16 years]), resulting in a total of 2268 training images. The independent holdout test dataset included 175 examinations of 174 patients (median age at first acquisition: 50 years [IQR: 16 years]), resulting in 350 test images. No significant difference in the distribution of the age could be observed between the training cohort and the test cohort, neither when including only the first examination of each patient (*p* value: 0.66), nor when also including repeated studies (*p* value: 0.91).

**Table 1:** Demographic data, sample characteristics, and target class distribution across the training dataset and the independent holdout test dataset.

Variable	Overall sample	Training dataset	Test dataset
N patients	1158	984	174
*Age*			
Median age (IQR) [years]	50 (17)	50 (17)	50 (16)
Median age (IQR) at first acquisition [years]	50 (16.75)	50 (16)	50 (16)
N examinations	1309	1134	175
*N repeated examinations per patient*			
One examination	1020	847	173
Two examinations	125	124	1
Three examinations	13	13	0
*N images*	2618	2268	350
Left breast	1309	1134	175
Right breast	1309	1134	175
*N artifacts (%)*	961 (37%)	777 (34%)	184 (53%)
Left breast	466 (36%)	379 (33%)	87 (50%)
Right breast	495 (38%)	398 (35%)	97 (55%)

*IQR* interquartile range.

### Interrater agreement

Regarding individual images, the interrater agreement between the three independent observers was Kappa = 0.577 (*p* < 0.001), corresponding to a moderate agreement according to Landis and Koch^20^. The interrater agreement between the three observers stratified by laterality was Kappa = 0.573 (*p* < 0.001) for images of the left breast and Kappa = 0.579 (*p* < 0.001) for images of the right breast.

### Artifacts on DWI sequences

According to the visual artifact assessment by the three observers, artifacts were present in 37% (961 out of 2618 images) of all images in the dataset. When considering both regions of interest (ROIs) together for each MRI examination, artifacts were present bilaterally in 26% (340), whereas unilateral artifacts occurred in 21.5% (281) of the examinations and a total of 52.6% (688) examinations were free from artifacts.

In the training dataset, artifacts were present in 34% (777 out of 2268 images), whereas in the test dataset, artifact prevalence was 53% (184 out of 350 images) of all images, corresponding to a statistically significant difference (*p* value: < 0.001).

### Artifact detection using deep learning

**Figure 1:**
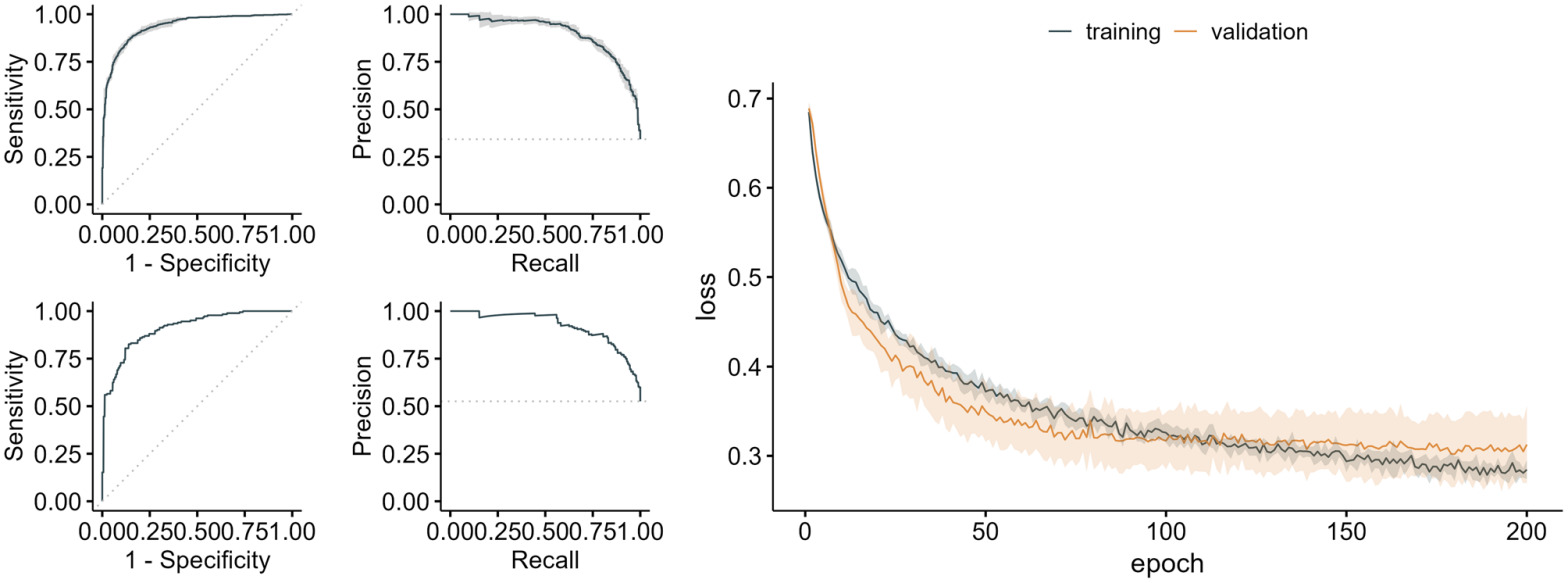
Deep learning results. The figure shows the receiver operating characteristic (ROC) curve (left column) and the precision-recall (PR) curve (mid column) and the loss curves (right column) for the DenseNet architecture. Row 1: ROC and PR curve averaged over the 5 cross-validation folds. Row 2: ROC and PR curve for the ensemble’s prediction on the independent holdout test dataset. The training loss (dark blue) and the validation loss (yellow) curves are averaged over 5 CV folds.

Figure 1 shows the receiver operating characteristic (ROC) curve (left column) and the precision-recall (PR) curve (mid column) of the resulting DenseNet models from the fivefold cross-validation (CV) (row 1). Row 2 of Fig. 1 shows the corresponding performance curves of the ensemble of the 5 CV models computed using the predictions for the holdout test dataset. The training and validation loss curves for the models averaged over the 5 CV folds are shown in the right column of Fig. 1. The training performance measures for each model from the 5 CV-folds as well as the corresponding best epochs are given in supplemental Table S2. All 5 CV models were applied to predict the outcome in the independent holdout test dataset. On average, the DenseNet achieved an area under the PR curve of 0.915 (± 0.004) with a positive predictive value (PPV) of 0.953 (± 0.013) and a specificity of 0.970 (± 0.008) for the detection of significant artifacts on breast DWI MIPs in the independent holdout test dataset (Table 2). The DenseNet ensemble—created by calculating the arithmetic mean of the predicted probabilities of the 5 models for each image in the holdout test dataset and considering images with an averaged probability of $>0.5$ to contain artifacts—showed an area under the PR curve of 0.921, with a PPV of 0.981 and a Specificity of 0.988, respectively (Table 2, column 8).

**Table 2:** Holdout test dataset performance.

Variable	M1	M2	M3	M4	M5	Mean (SD)	DenseNet ensemble
Accuracy	0.746	0.757	0.737	0.760	0.746	0.749 (± 0.009)	0.763
AUROC	0.904	0.910	0.906	0.895	0.900	0.903 (± 0.006)	0.910
AUPRC	0.917	0.921	0.914	0.912	0.912	0.915 (± 0.004)	0.921
Sensitivity	0.538	0.571	0.533	0.560	0.549	0.550 (± 0.016)	0.560
Specificity	0.976	0.964	0.964	0.982	0.964	0.970 (± 0.008)	0.988
PPV	0.961	0.946	0.942	0.972	0.944	0.953 (± 0.013)	0.981
NPV	0.656	0.669	0.650	0.668	0.658	0.660 (± 0.008)	0.669

The table shows the performance measures of the 5 DenseNet cross-validation (CV) models (columns 2–6) along with their averaged performance (column 7) when applied to predict the outcome in the independent holdout test dataset (n = 350 images). Column 8 shows the performance of the DenseNet ensemble. The ensemble was created by calculating the arithmetic mean of the predicted probabilities of the 5 models for each image in the holdout test dataset and considering images with an averaged probability of $>0.5$ to contain artifacts. Mean: (unweighted) average over 5 CV folds. SD: (unweighted) standard deviation over 5 CV folds. *AUROC* area under the ROC curve, *AUPRC* area under the precision-recall curve, *PPV* positive predictive value, *NPV* negative predictive value.

**Figure 2:**
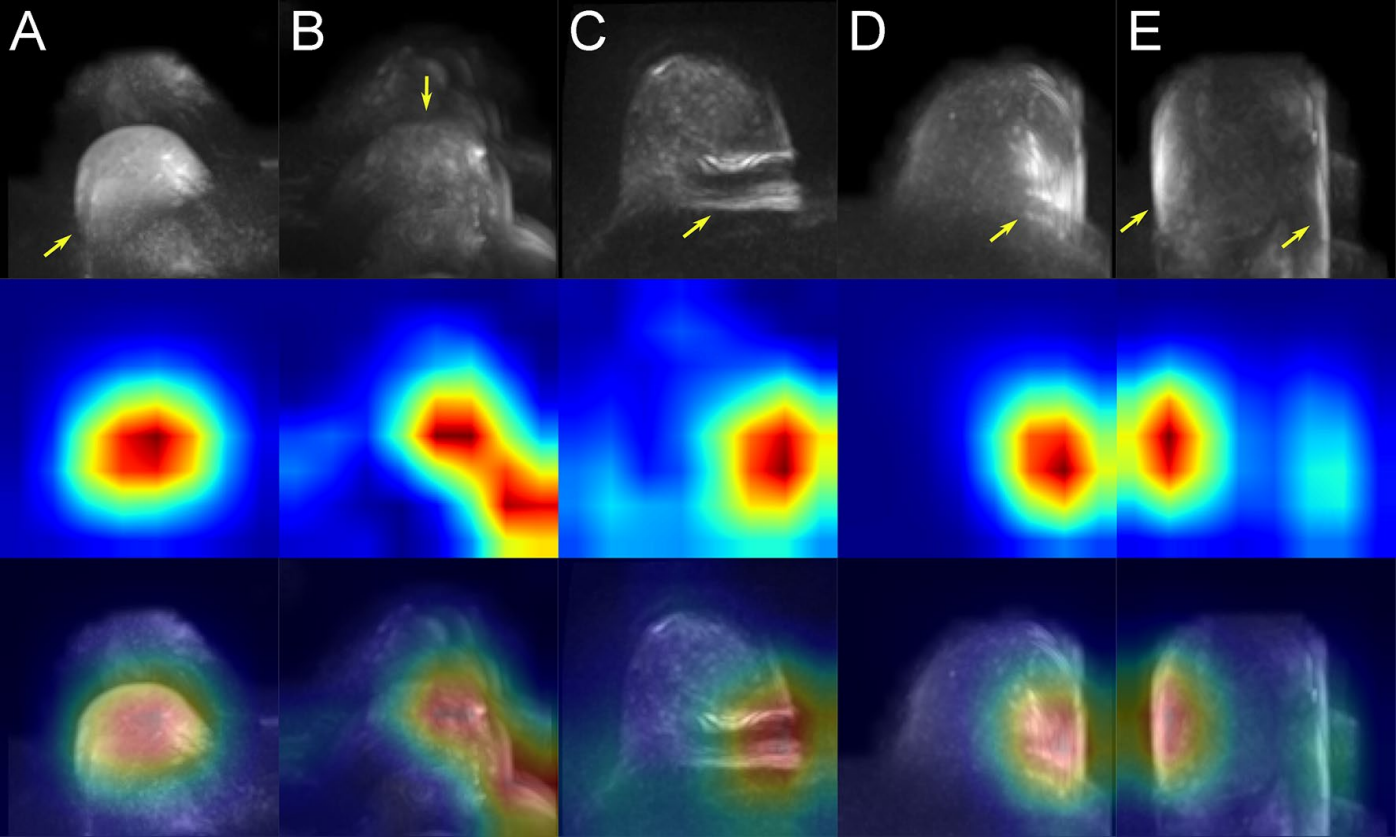
Class activation maps (examples): true positives. Original images are shown in row 1 (**A**–**E**). The Grad-CAM++ visualization for the predicted class (i.e. prediction/ground truth = ‘artifact’) are shown in row 2 and images of row 3 show the combined images. The heatmaps’ color gradient shows from blue to red the relevance of each pixel for the inference of the respective class. Artifacts in DWI often originate from multiple technical and/or patient-related sources that may be interdepend and thus it is not always possible to attribute one specific artifact source. The arrows mark regions of artifacts within the images with possible contributing factors of insufficient fat suppression (e.g. visible in **A**), ghosting artifacts related to silicone implants (e.g. **B**), artifacts related to combinatory effects of distortion and insufficient fat suppression (e.g. visible in **C**) and related to remaining surface coil flares (e.g. visible in **D**, **E**).

**Figure 3:**
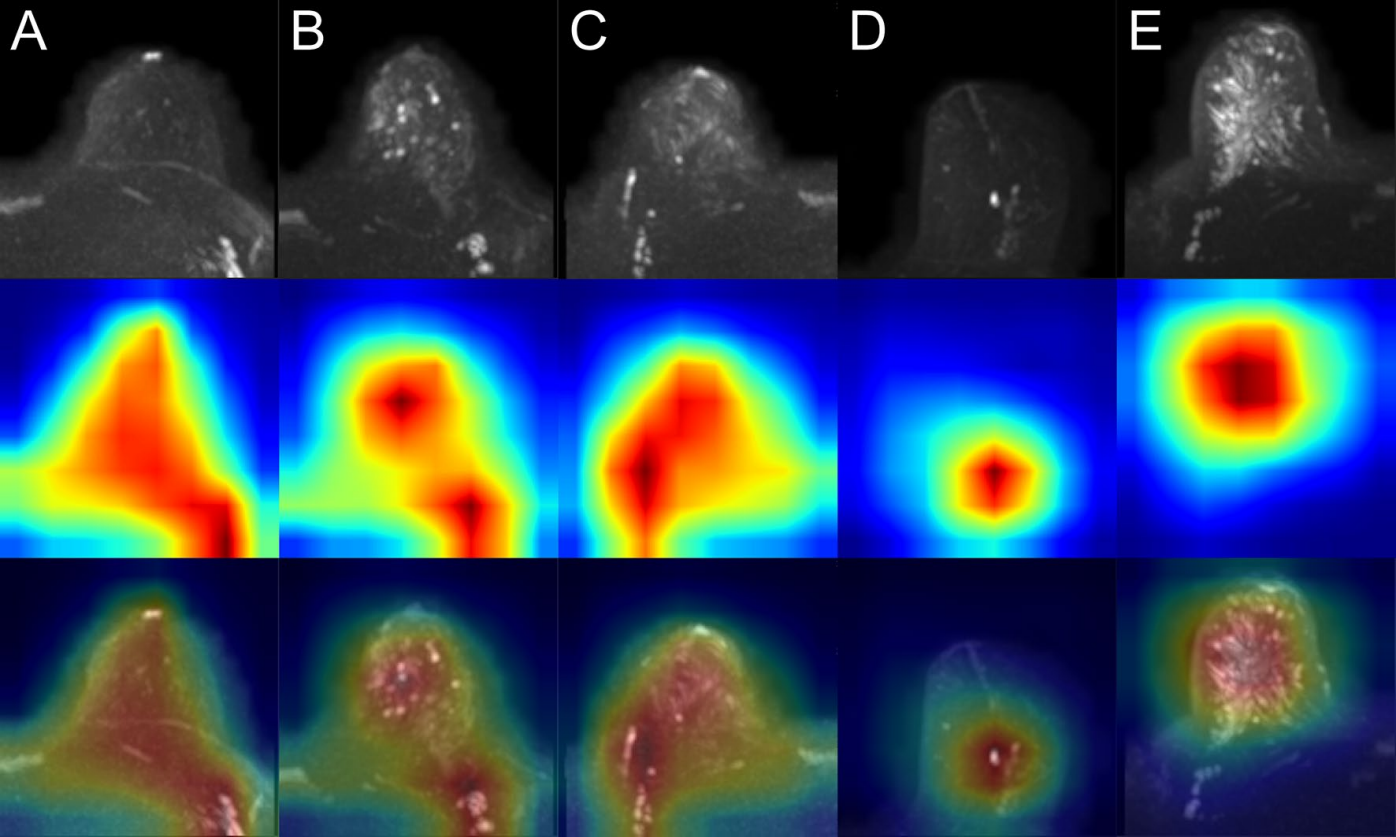
Class activation maps (examples): true negatives. Original images are shown in row 1 (**A**–**E**). The Grad-CAM++ visualization for the predicted class (i.e. prediction/ground truth = ‘artifact-free’) are shown in row 2 and images of row 3 show the combined images. The heatmaps’ color gradient shows from blue to red the relevance of each pixel for the inference of the respective class.

**Figure 4:**
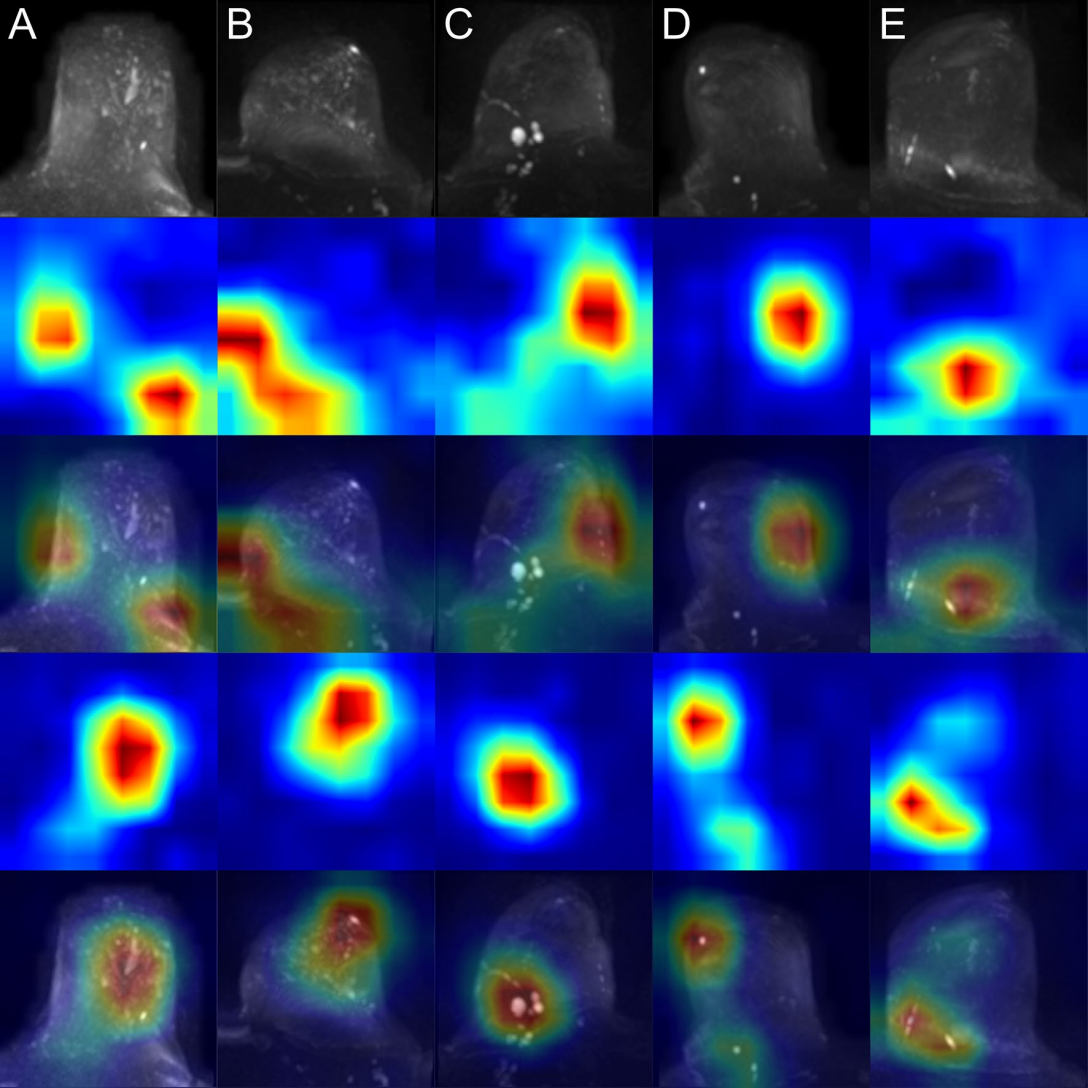
Class activation maps (examples): false positives. Original images are shown in row 1 (**A**–**E**). Rows 2–3 show the Grad-CAM++ visualization and the combined image for the predicted class (i.e. prediction = ‘artifact’). Rows 4–5 show the Grad-CAM++ visualization and the combined image for the actual class (i.e. ground truth = ‘artifact-free’). The heatmaps’ color gradient shows from blue to red the relevance of each pixel for the inference of the respective class. A detailed interpretation of the original images and Grad-CAM++ visualizations of the (falsely) predicted class (rows 2–3) showed indeed corresponding areas of slight blurry and/or hyperintense appearing image regions, which, however, were rated as not significant by ≥ 2 out of three independent raters.

**Figure 5:**
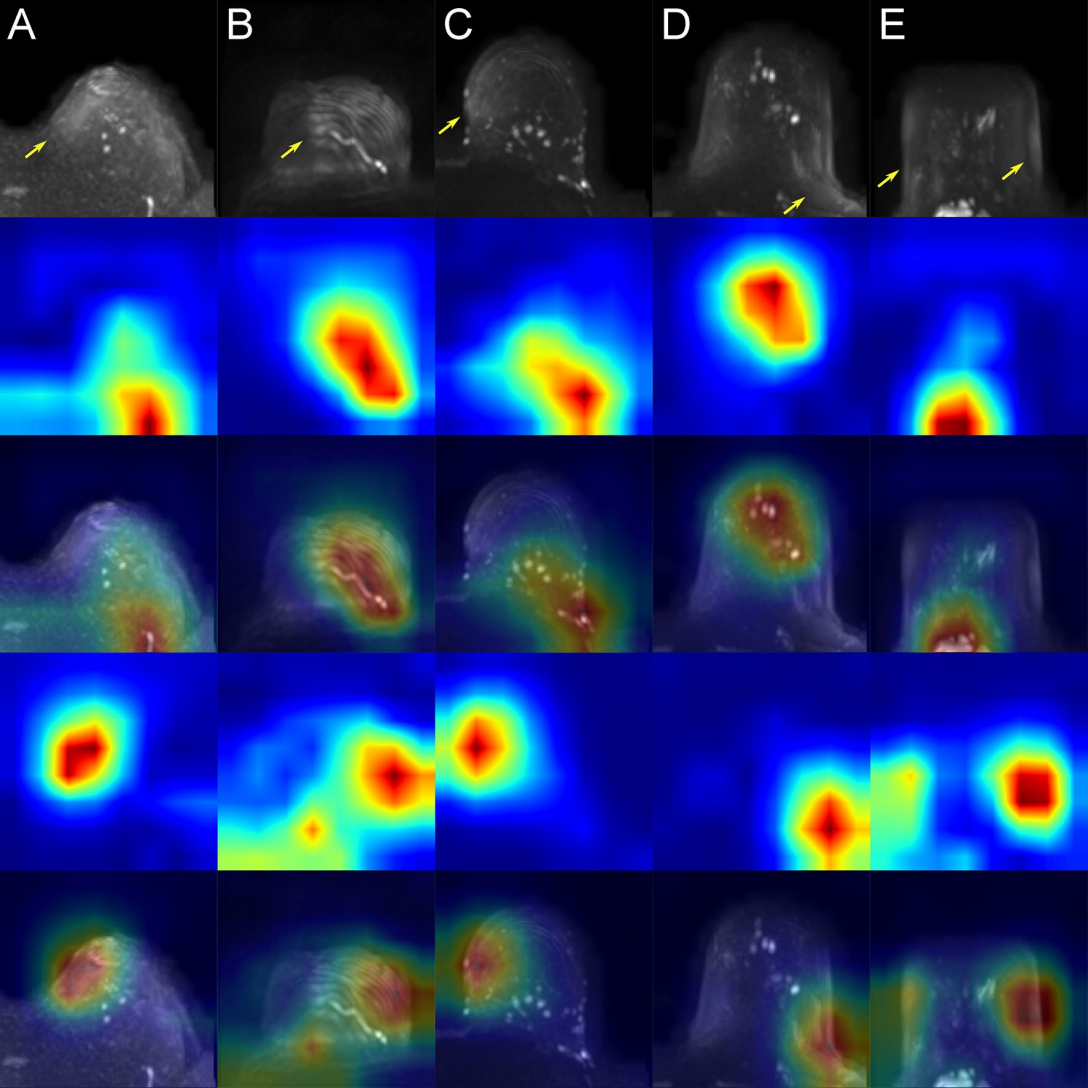
Class activation maps (examples): false negatives. Original images are shown in row 1 (**A**–**E**). Rows 2–3 show the Grad-CAM++ visualization and the combined image for the predicted class (i.e. prediction = ‘artifact-free’). Rows 4–5 show the Grad-CAM++ visualization and the combined image for the actual class (i.e. ground truth = ‘artifact’). The heatmaps’ color gradient shows from blue to red the relevance of each pixel for the inference of the respective class. Artifacts in DWI often originate from multiple technical and/or patient-related sources that may be interdepend and thus it is not always possible to attribute one specific artifact source. The arrows mark regions of artifacts within the images with possible contributing factors of insufficient fat suppression (e.g. visible in **A**), artifacts emerging in the MIP corresponding to the repetition of thickened cutis projected into MIP (no technical artifact) (as visible in **B** and **C**, with the latter including artifacts of insufficient fat suppression), and artifacts associated to remaining surface coil flares (e.g. visible in **D**, **E**).

Examples of class activation maps (CAMs) for true positive, true negative, false positive and false negative predicted images are shown in Figs. 2, 3, 4 and 5. The CAMs were computed using the model with the highest area under the PR curve during training (i.e. CV fold 5, AUPRC = 0.911; see supplemental Table S2). The GradCAM++-results suggest that the network is capable to detect artifacts well (Fig. 2). From the generated CAM images can also be derived that in the absence of high signal intensities in the breast tissue, the whole organ seems to contribute to the class assignment for correctly classified artifact-free images, whereas in the presence of high signal intensities, the most important class-discriminative regions seem to correlate with areas that include blood vessels and fibroglandular breast tissue (FGT) (Fig. 3). The latter observation is quite in line with our previous results for the artifact detection in MRI-derived DCE-MIPs where a sharp demarcation of contrast agent-containing blood vessels from the surrounding breast tissue was considered to guide the neural network (NN) towards the negative class (both, true and false negative)^21^. For DWI MIPs, in addition to the lack of contrast agent administration, the demarcation of blood vessels and FGT is not as clear and sharp as in DCE MIPs. Nevertheless, the CAM results for the DWI MIPs let us assume that the mentioned attributes could be features used by the NN to distinguish between artifact-free and artifact-containing images. This is further underlined by the CAM results of the false negative classifications (Fig. 5), where image regions with high intensity values, such as areas containing blood vessels or FGT, seem to have guided the NN towards its (false) decision (i.e. falsely classifying them as artifact-free; rows 2–3 in Fig. 5), overseeing artifacts present in other image regions (yellow arrows, and rows 4–5 in Fig. 5). In contrast, the most important class-discriminative regions for false positive classifications seem to correlate with slightly blurry appearing image regions (rows 2–3 in Fig. 4). When providing the corresponding ground truth (i.e. artifact-free) to the computation of the CAM images, the class-discriminative regions are again overlapping with regions that contain blood vessels and FGT (rows 4–5 in Fig. 4).

**Figure 6:**
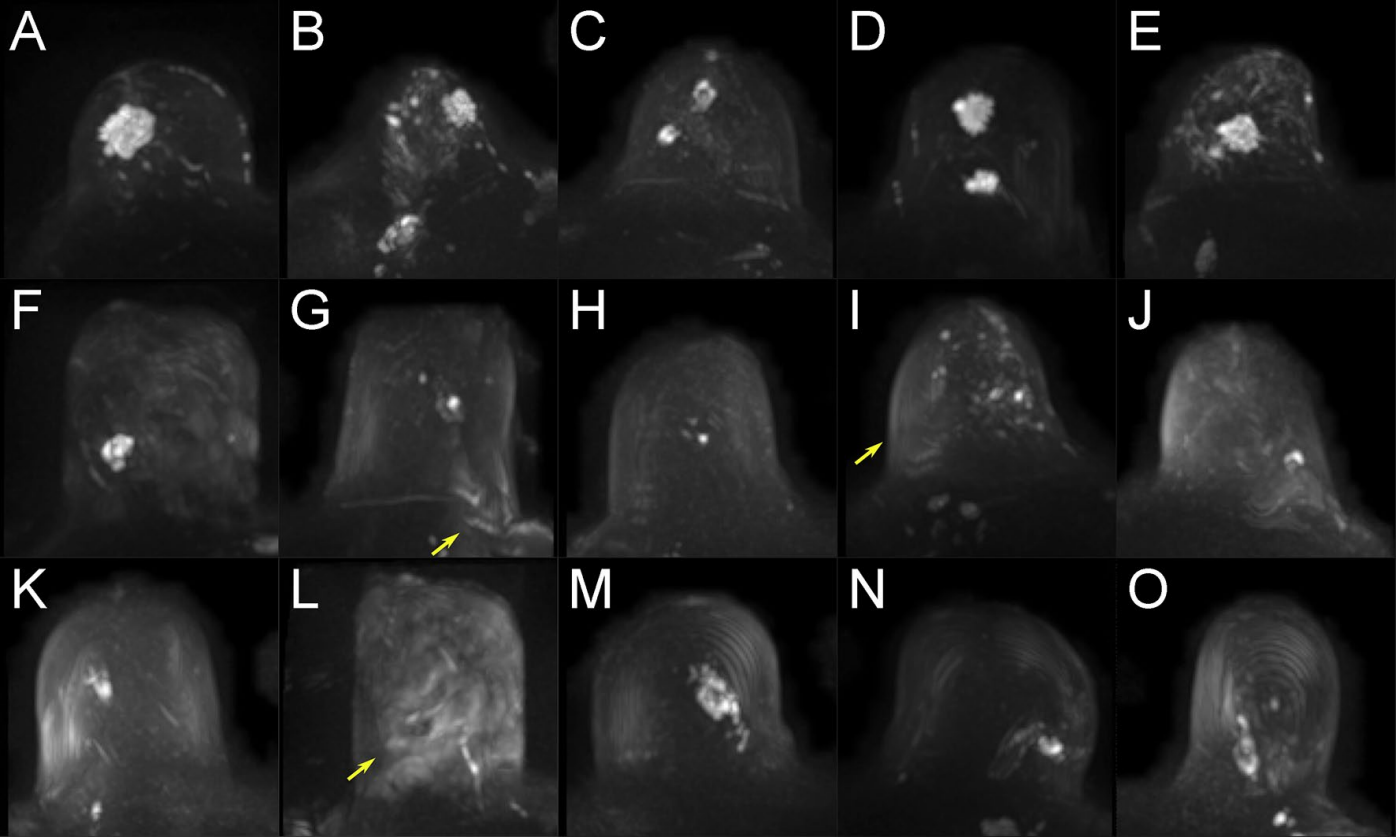
BI-RADS 6 lesions in clinical cases (examples). Each tile of the figure presents the left or right breast of one clinical case with a diagnosed BI-RADS 6 lesion. Row 1 (**A**–**E**) shows images without the presence of artifacts. Row 2 (**F**–**J**) shows images that contain artifacts with no or moderate influence on the diagnostic assessment. Row 3 (**K**–**O**) shows images with artifacts that significantly impede the diagnostic evaluation. Artifacts in DWI often originate from multiple technical and/or patient-related sources that may be interdepend and thus it is not always possible to attribute one specific artifact source. The arrows mark regions of artifacts within the images with possible contributing factors related to distortion (e.g. visible in **G**), insufficient fat suppression (e.g. visible in **I**, **L**), related to remaining surface coil flare (e.g. also visible in **I**), and pulsation related signal drops in DWI (e.g. also visible in **L**).

Figure 6 shows DWI MIP ROIs of 15 clinical cases with BI-RADS 6 lesions from our dataset with various gradations of artifacts. Images A–E represent 5 cases without MRI artifacts. Images F–J are examples, where present artifacts have a moderate influence on the diagnostic evaluation, whereas images K–O contain examples with artifacts that would significantly impede a diagnostic evaluation. A corresponding graphic with BI-RADS 5 lesion is given in supplemental Fig. S1.

## Discussion

Here we demonstrated the capability of an NN to detect MRI artifacts on qualitative DWI-derived MIPs. The DenseNet was trained on more than 2200 images of 1134 individual MRI examinations. The ensemble of the 5 CV models showed an area under the PR curve of 0.921 on the independent holdout test dataset with a PPV of 0.981 and a Specificity of 0.988, respectively. These results indicate that the ensemble classifier was able to detect artifact-containing images in the test dataset quite well.

MRI examinations of the female breast increasingly include DWI in the sequence protocol. DWI allows to detect suspicious focal and non-focal alterations of tissue diffusivity and to provide quantitative measures of derived parameters such as the apparent diffusion coefficient (ADC). One advantage of DWI is that it does not require the application of gadolinium containing intravenous contrast agents. The relation of gadolinium containing contrast agents to findings of deposition in the human body as well as in the environment has been under investigation over the past years^11–14, 22–25^. This has intensified the research on non-contrast enhanced MRI techniques of which DWI is of special interest. With the potential to perform a DWI MRI in only a couple of minutes and to avoid both ionizing irradiation and the application of contrast agents of any kind, DWI has also been investigated in breast MRI both as an expansion of DCE MRI and as a possible stand-alone application in the context of breast cancer screening^5–8^.

Whilst the potential of DWI has been demonstrated in several studies, the sequence remains technically challenging and prone to artifacts^17^. This can impede the diagnostic evaluation of DWI images, which is further aggravated when image postprocessing techniques are applied, such as the generation of DWI derived MIPs for initial diagnostic assessment. The use of MIPs in breast MRI has largely evolved based on a publication by Kuhl et al. demonstrating the capability of DCE MRI derived MIPs to provide a high diagnostic accuracy while simultaneously reducing the reading times for radiologists^9^. Similar to DCE MRI, DWI also offers the possibility of generating MIPs from high b-value acquisitions that provide sufficient suppression of the FGT, leaving mainly the areas of potential interest visible in the image. Feasibility studies already demonstrated a high diagnostic accuracy when using the combination of high b-value DWI and MIPs in the initial reading of breast MRI^16, 18^. In general, reading schemes that involve MIPs depend on a high image quality to a large extent, because artifacts could potentially cover suspicious lesions on the 2D image and MIPs are particularly prone to artifacts as hyperintense artifacts may accumulate from the single slices into the MIP image. In the context of DWI, the generation of MIPs can be technically challenging as DWI itself is prone to image artifacts, even if the basic sequence has intensively been adjusted in order to avoid them.

Especially in the context of abbreviated MRI protocols, high image quality is particularly important since complementary image sequences might not be available to compensate for potential artifacts. In our dataset, artifacts were present in almost half of all examinations. Most common sources of artifacts include patient movement and insufficient fat suppression. Both types of artifacts can be difficult to avoid in advance, so an immediate assessment of image quality—potentially running directly on the MRI machine—could be of great importance, for example, to trigger actions that allow further handling of images with artifacts or even to repeat acquisitions. For example, the artifact detection algorithm could be implemented in a setting in which reading protocols include the initial assessment of a MIP image, such as abbreviated MRI protocols applied in a breast cancer screening context. During the image post-processing, the NN could label or select MIPs with sufficient image quality to be presented to the radiologist, as for artifact-containing MIPs it would mostly be unnecessary to open the MIP and instead single slice sequences could be read directly. Furthermore, hypothetically, the artifact-detection could also be applied during the ongoing examination and in the case of a poor image quality, for example, the acquisition of the DWI sequence could be repeated. One could also imagine to acquire DCE sequences only in the case of a detected poor image quality on the DWI MIP, however, all of these scenarios were not evaluated prospectively in our study. In addition to its application in clinical workflows, such an algorithm could also be used to detect artifacts in the curation and preparation of datasets for deep learning tasks, such as the automated detection of breast lesions.

In this feasibility study, we focused on applying the artifact detection directly on 2D MIP images, as they are a diagnostic tool increasingly investigated for its diagnostic value in abbreviated study protocols, and as outlined above, MIPs harbor a particular risk for artifact representation. The DenseNet NN architecture used in this study has also been applied previously by our group to detect MRI artifacts on DCE MIPs^21^. While the NN achieved a high area under the PR curve averaged over the 5 CV folds (supplemental Table S2) for detecting artifacts in DWI MIPs, not all artifacts were correctly classified and false positives occurred, especially in case of image regions that appeared blurred, as shown in the example CAM images in Fig. 4. Strictly speaking, the network here indeed detected slight artifacts in images with negative class labels, which, however, were rated as not significant by $\ge 2$ out of the three independent raters.

The issue of MRI associated artifacts is well known and different solutions exist to address, for example, patient motion in MRI, which are summarized in a review article by Zaitsev et al.^26^. Common mitigation strategies for motion artifacts include, for example, *motion prevention* (e.g. training, breathhold, sedation in case of patients not able to comply with instructions such as children, etc.). *artifact reduction* (e.g. faster imaging, phase reordering, etc.), and *motion correction* (e.g. navigators, pro-/retrospective correction)^26^. Commonly employed post-processing techniques for motion correction include image registration, which aims at ensuring spatial alignment of separate images^27^ and several algorithms have been adapted to breast MRI (e.g.^28–31^). While all of these approaches aim to improve the image quality of the acquired scan data, the NN presented here can be considered as a complementary method that could capture the remaining artifacts in MIPs after the aforementioned methods have been applied.

Our study has several limitations. First, the major limitation is the binary labeling used this study as artifacts can occur with a wide spectrum in terms of their severity and subsequent clinical relevance, which is most likely not represented satisfactorily by two classes. A multi-reader assessment with three independent raters and a best-of-n approach to define the target label was performed in order to establish a reliable ground truth for the presence of artifacts. However, the interrater agreement with a Kappa-statistic of 0.58 in our study—corresponding to a moderate strength of agreement according to Landis and Koch^20^—indicates certain challenges associated with artifact classifications in novel imaging techniques. Therefore, the development of acknowledged and objective assessment criteria to rate the severity of MRI artifacts would be of high interest, which would also allow for a repetition of our study with a more finely granulated labeling regarding artifact severity. Second, variations in the DWI sequence settings between the different MRI examinations resulted in heterogeneous image quality. However, this was not further explored in our study, and therefore the results are not suitable to make statements neither regarding the relationship between sequence settings and artifact susceptibility nor about the influence of the sequence settings on the artifact detection performance of the NN. Third, we only used the MIPs as input for the NN, so that the influence of other variables on the automated artifact detection, e.g. scanner-related features or demographic characteristics remains unclear. Future studies could extract more features from the data, such as patient age, body-mass-index, breast size, breast density, and patient age, and include these into the model building process as well. Furthermore, our study was performed using 2D DWI MIPs, which are currently of subordinated clinical relevance as compared to the 3D DWI sequences. As our study was intended as a preparatory groundwork to investigate the capability of a CNN to identify artifacts on DWI images, we considered the artifact detection on MIPs as a good starting point. Furthermore, considering the proneness to artifacts of DWI combined with the potential aggravation of artifacts when computing MIPs, we identified this imaging post-processing technique as one that would probably benefit strongly from an artifact detection algorithm, especially when using MIPs for the initial reading, e.g., as in abbreviated MRI protocols. However, future work should investigate and evaluate artifact detection on 3D DWI sequences as well and compare the results with the detection on MIPs and perhaps identify other potential areas of application. Future work in this field is important especially since MIPs accumulate hyperintense artifacts, whereas hypointense artifacts may go unnoticed since probably not being detectable due to the basic technical principle of MIPs. Another limitation is that there was no stratification on a patient level when creating the CV folds, potentially leading to images of the same patient being present in both, the CV folds’ training and validation dataset. In theory, this could lead to an overly optimistic validation error if one would assume that the network would learn features from a MIP of a patient from the training dataset, which would help to better infer the artifact-class of either the contralateral breast or a MIP from another examination of the same patient that could potentially be included in the corresponding validation dataset. However, DWI artifacts originate mostly from technical issues (such as poor shimming, insufficient fat saturation, magnetic susceptibility differences or eddy currents^32^) or from patient movement, which are rather examination-specific features than dependent on patient characteristics and thus, such a stratification might not be important for the task at hand. To address this potential issue, an independent holdout test dataset was formed to get an unbiased final model evaluation. This test dataset contained only new patients that were not yet available in the training dataset. For the final model evaluation, the trained models were applied to predict the artifact class in this independent holdout test dataset. The consistency of the model performance results between the CV-training and the evaluation on the independent holdout test dataset indicates that the NN was indeed capable of learning features that are related to MRI artifacts on DWI-MIPs of the breast. Another restriction of this study is imposed by the use of high b-value DWI series. It remains unclear as by this study to what degree the results might be achievable as well at lower b-values. Furthermore, we did not have the possibility to apply our approach across different MRI vendors. Thus, future studies are needed to assess the aspect of generalizability of this algorithm to images of different b-values, image quality, and scanner systems. Last but not least, another limitation could be that the dataset represents a retrospective university hospital patient cohort and thus we cannot state to what degree similar patient- and artifact characteristics might be found, e.g., in a screening population and whether using such an artifact detection algorithm in clinical routine would actually lead to improvements in the reading process.

In conclusion, we here demonstrated an NN that detects artifacts in breast DWI-derived MIPs. The network was able to identify artifact-containing images in the independent holdout test dataset quite well and might serve as a starting point to develop more sophisticated quality assurance methods for breast MRI DWI sequences in the future.

## Materials and methods

### Study sample and ethics approval

This retrospective study included breast MRI examinations from March 2017 to June 2020. The study was approved by the ethics committee of the Friedrich-Alexander-University (FAU) Erlangen-Nürnberg, waiving the need for informed consent. The authors declare that this research was performed in compliance with the World Medical Association Declaration of Helsinki on Ethical Principles for Medical Research Involving Human Subjects. Inclusion criteria were the acquisition of a clinically indicated breast MRI at the Institute of Radiology of the University Hospital Erlangen (UHE) with at least one DWI sequence that was acquired with a high b-value equal to 1500 s/mm^2^. For eligible examinations, additional series derived from the originally acquired DWI sequence (e.g. motion-correction or otherwise post-processed series) were excluded from the dataset. This cohort is partially overlapping with a previously reported study sample, in which we investigated the automated detection of artifacts using deep learning on DCE sequences^21^. However, the previous work included data from the years 2015 to 2019 and the DWI sequences have not been assessed there.

### MRI protocol

All MRI examinations were performed with a clinical indication at the hospital’s routine MRI scanners (1.5 and 3 Tesla MRI; Model names: Magnetom Aera, Vida, and Skyra from Siemens Healthineers, Erlangen, Germany). The routine MRI protocols consisted of morphologic, T2-weighted, dynamic contrast-enhanced T1-weighted, and DWI sequences. The standard positioning of patients during the examination was in prone position with arms laterally to the body. DWI acquisitions were performed using different sequence types and ranges of b-values, commonly including b = 0, b = 750, and b = 1500 s/mm^2^. Supplemental Table S1 gives a detailed overview of the different settings on which the DWI sequences of this study were based.

### Data processing

Imaging data were queried from the local routine picture archiving and communication system and transferred to evaluation workstations within the UHE Institute of Radiology. The DWI MRI sequences were processed in a similar manner as previously described for the DCE sequences^21^. An in-house developed Python script was used to represent the voxels with the highest intensity values along the z-axis (i.e. head-feet direction) on a transversal 2D image in order to compute a MIP from each individual qualitative DWI sequence. Quadratic tiles with a dimension of $1/2\hspace{0.25em}imag{e}_{width}\times 1/2\hspace{0.25em}imag{e}_{width}$ containing the left and right breast as ROIs were cropped out from the upper left and right parts of each MIP and saved as JPEG files for the visual artifact assessment by the three observers.

### Visual artifact assessment

All processed images were labeled in binary manner by three independent observers (S.B., E.L.B., L.A.K.) with regard to the presence of significant artifacts (1 = artifacts present; 0 = no artifacts present). Artifacts on the DWI MIPs were visually evaluated, with sources and possible characteristics of artifacts derived from recent literature as found, e.g., in Partridge et al.^33^. The rating was performed regardless of whether or not the specific artifacts covered a significant breast lesion. All artifacts that could mask a lesion were therefore considered significant, regardless of the actual clinical relevance in the individual examination. A final label was computed for each image using the *best-of-n* approach, i.e., if $\ge 2$ raters classified an image as to be of the positive class, the final label of the image was “artifacts present”. This final label was used for the subsequent experiments and data analyses.

### Image preprocessing and image augmentation

Image preprocessing was performed in Python (version 3.8.5) using *SimpleITK* version 2.0.2^34^ following the procedure as previously described^21^. From each DWI volume, a MIP was computed as described above. To preprocess the cropped ROIs for training the deep learning networks, the images were further normalized (mean = 0, standard deviation = 1), resized to $256\times 256$ pixels, and saved as *NumPy* arrays^35^. Image augmentation included random rotation (probability: 0.5, maximum angle: 180 degrees), random flip across x-axis and y-axis (probability: 0.5) and random zoom (probability: 0.5, minimum zoom: 0.5, maximum zoom: 1.5).

### Deep learning

A DenseNet121^36^ was trained to classify the presence or absence of artifacts on qualitative DWI MIPs of the left and right breast (i.e. a binary classification), utilizing the network architecture already implemented in the *monai* library version 0.4.0^37^, which builds upon the *PyTorch* deep learning framework version 1.7.1^38^. The code was further organized with *PyTorchLightning* version 1.2.4^39^, a wrapper for *PyTorch* that is tailored to application in research. The training of the deep learning network was carried out on a Tesla V100 graphical processing unit (GPU) with 32 GB memory and an Intel^®^ Xeon^®^ CPU E5-2698 v4 @2.20 GHz (20 cores) with 256 GB RAM. The methodology to carry out the experiments was aligned to our previous work^21^. A training dataset was formed from the examinations acquired up to and including the year 2019. An independent holdout test dataset set was formed from the examinations that were acquired in 2020 using only patients, which were not already included in the training dataset. The model parameters were optimized with a grid search on the training dataset, partitioning it by 80% to 20% for model training and evaluation (data not shown). Due to the observed class imbalance in the training dataset with the majority of images belonging to the negative class (i.e. no artifacts present), we primarily focused on the PR curve for evaluating the model performance, since it is said to more reliable in datasets with an imbalanced target class than the ROC curve^40^. The as such optimized model parameters were validated with a fivefold CV on the training dataset. The CV folds were generated in a stratified manner to ensure a similar artifact prevalence across the folds. For each fold, the training data was further randomly split into 80% that were actually used for training and 20% that were used for validation, i.e., monitoring loss and performance metrics during network training. Binary cross entropy with logits from *PyTorch* was employed as loss function. Class probabilities were calculated using the softmax function. We employed the ‘Adam’^41^ optimizer with a weight decay of $1{e}^{-5}$ and an initial learning rate of $\eta =3{e}^{-5}$. The DenseNet121 network was further parameterized with a dropout probability of 10%. All models were trained for 200 epochs with a batch size of 128, resulting in 12 steps per epoch. For each CV model, the weights from the epoch with the lowest validation loss observed within 200 epochs were chosen to predict the respective CV fold’s validation dataset and the observations in the independent holdout test dataset. In accordance with our previous work^21^, we also created an ensemble from the 5 CV models to predict the artifact presence in the independent holdout test dataset. The ensemble was created by calculating the arithmetic mean of the predicted probabilities of the 5 CV models for each image in the test dataset and considering images with an averaged probability of $>0.5$ to contain artifacts.

### Statistical analysis

The statistical analyses were performed with the R software version 4.2.1^42^. Summary statistics were computed in base R^42^. Fleiss’ Kappa^43^ was computed to test for interrater agreement of the artifact assessment between the three raters using the R package *irr* version 0.84.1^44^. Model metrics were calculated using the *mlr3measures* package version 0.5.0^45^. Graphics were created with the R packages *ggplot2* version 3.3.6^46^, *ggpubr* version 0.4.0^47^ and *precrec* version 0.12.9^48^. Wilcoxon’s rank sum test (two-sided)^49, 50^ was used for comparing the distribution of continuous variables between two groups. Differences between two categorical variables were assessed with the Chi-squared test^51^. Significance level was set to $\alpha$=0.05. No correction for multiplicity was performed. Class activation maps (CAMs) depict so-called class-discriminative regions, which can be displayed as heatmaps that color-code image regions that are deemed important by the CNN classifier to identify the inferred class^52^. This method builds upon the finding that components of CNNs inherently have object detection capabilities^53^ that allow to efficiently localize discriminative regions in the image, e.g. the pixels that discriminate between the categories a classifier was trained with^52^. The CAMs were generated for all images from the independent holdout test dataset, using the model that achieved the highest area under the PR curve during the fivefold CV. The CAM images were computed with the GradCAM++ algorithm^54^ provided with the *monai* library^37^ version 0.4.0, and the resulting images were assessed visually by an experienced board-certified radiologist (S.B.).

## Supplementary Information

Supplementary Information.

## Data Availability

The datasets generated and/or analyzed during the current study are not publicly available due to internal data transfer policies but are available from the corresponding author on reasonable request.

## Abbreviations

ADC: Apparent diffusion coefficient
AI: Artificial intelligence
AUPRC: Area under the precision-recall curve
AUROC: Area under the receiver operating characteristic curve
CAM: Class activation map
CNN: Convolutional neural network
CPU: Central processing unit
CV: Cross validation
DCE: Dynamic contrast enhanced
DWI: Diffusion weighted imaging
FGT: Fibroglandular breast tissue
GB: Gigabyte
GPU: Graphics processing unit
IQR: Interquartile range
IRB: Institutional review board
MIP: Maximum intensity projection
Mm: Millimeter
MRI: Magnetic resonance imaging
NPV: Negative predictive value
PPV: Positive predictive value
PR: Precision-recall
RAM: Random-access memory
ROC: Receiver operating characteristic
ROI: Region of interest
s: Seconds
T: Tesla
UHE: University Hospital Erlangen

## Code availability

The code used to perform the experiments is available at GitHub: https://github.com/kapsner/dwi_mip_artifact_classifier.
