# Supplementary material for: Image quality assessment using deep learning in high b-value diffusion-weighted breast MRI
Source: Sci Rep. 2023 Jun 29;13:10549. doi: 10.1038/s41598-023-37342-3 (PMC10310703; doi:10.1038/s41598-023-37342-3)
Supplement: Supplementary file 1 — Supplementary Information. [file 41598_2023_37342_MOESM1_ESM.pdf]

Image Quality Assessment Using Deep Learning in High b-Value Diffusion-Weighted Breast MRI

Lorenz A. Kapsner (M.D.)[1,2,*], Eva L. Balbach (M.D.)[1], Lukas Folle (M.Sc.)[3], Frederik B. Laun (Dr. rer. nat.)[1], Armin M. Nagel (Dr. rer. nat.)[1], Andrzej Liebert (Dr. hum. biol.)[1], Julius Emons (M.D.)[4], Sabine Ohlmeyer (M.D.)[1], Michael Uder (M.D.)[1], Evelyn Wenkel (M.D.)[1], and Sebastian Bickelhaupt (M.D.)[1,5]

[1] Institute of Radiology, Universitätsklinikum Erlangen, Friedrich-Alexander-University Erlangen-Nürnberg (FAU), Maximiliansplatz 3, 91054 Erlangen, Germany.

[2] Medical Center for Information and Communication Technology, Universitätsklinikum Erlangen, Krankenhausstraße 12, 91054 Erlangen, Germany.

[3] Pattern Recognition Lab, Friedrich-Alexander- University Erlangen-Nürnberg (FAU), Martensstraße 3, 91058 Erlangen, Germany.

[4] Department of Obstetrics and Gynaecology, Universitätsklinikum Erlangen, Friedrich-Alexander-University Erlangen-Nürnberg (FAU), Universitätsstraße 21-23, 91054 Erlangen, Germany.

[5] German Cancer Research Center (DKFZ), Im Neuenheimer Feld 280, 69120 Heidelberg, Germany.

[*] Correspondence: Lorenz A. Kapsner (M.D.) <lorenz.kapsner@uk-erlangen.de>

1. Supplementary Information

1.1. MRI settings

Table S1 gives a detailed overview of the MRI settings for DWI sequences on which the images used in this study are based. All sequences were acquired in transversal orientation. All images were acquired with single-refocused single-shot diffusion-weighted echo planar imaging (EPI) sequences. Eddy currents effects were minimized with the vendor-provided “dynamic field correction” method. A short-tau inversion recovery fat saturation was used. Parallel imaging was performed with the Grappa algorithm (acceleration factor of 2, Grappa = GeneRalized Autocalibrating Partial Parallel Acquisition [1]). Most of the MRI examinations were performed using 3 Tesla (T) MRI scanners. The field of view (FoV) was in between 212×340 and 285×380 millimeters (mm) with a slice thickness of 4 mm. The echo time (TE) and repetition time (TR) ranged between 66 and 70 milliseconds (ms), and 6290 and 9660 ms, respectively. Examinations that were acquired with a 1.5 T Aera scanner provided the following acquisition parameters: Field of view (FoV) between 218×350 and 296×380 mm with a slice thickness of 3.5 to 5 mm, TE between 61 and 69 ms, and TR between 7770 and 12622.5 ms, respectively. At the Aera and Skyra fit scanners, a vendor-provided prototype sequence was used. It was equally susceptible to motion. Moreover, it was run also with a STIR fat saturation so that we deem it equally susceptible to motion and residual fat artifacts.

2. Supplementary Tables

Table S1: MRI protocols. MRI parameter settings of the diffusion weighted imaging (DWI) sequences that were related to the subtractions images used for creating the maximum intensity projections (MIPs). T: Tesla. TE: echo time. TR: time to repetition. TI: inversion time. ms: millisecond. mm: millimeter. [1]: TI not retrievable with DICOM tag ‘0018,0082’ for n=1 study. [2]: FoV not retrievable with DICOM tag ‘0051,100c’ for n=146 studies.

Model name	Magnetic field strength (T)	Sequence	b-values	Acquisition Matrix	FoV (mm)	TE (ms)	TR (ms)	TI (ms)	Slice thickness (mm)	N
Aera	1.5	Vendor-Provided Prototype EPI DWI	[50, 750, 1500], [50, 1500]	128 × 80 to 128 × 96	340 × 212 to 420 × 285	61 - 69	7770 - 12750	170	3.5 - 5	299
	1.5	EPI DWI	[50, 400, 1500], [50, 750, 1500]	128 × 80 to 164 × 108	350 × 218 to 350 × 230	83 - 123	8800 - 11000	180	4 - 5	3[1]
Skyra fit	3.0	Vendor-Provided Prototype EPI DWI	[50, 750, 1500]	128 × 80 to 128 × 100	350 × 218 to 450 × 296	66 - 70	6290 - 9660	220 - 250	4	630
Vida	3.0	EPI DWI	[50, 750, 1500]	128 × 80 to 128 × 100	350 × 218 to 400 × 273	66	6290 - 7800	250	4	377[2]

Table S2: Cross-validation results (model training). The table shows the performance measures of the 5 DenseNet cross-validation (CV) models on their validation datasets. Mean: (unweighted) average over 5 CV folds. SD: (unweighted) standard deviation over 5 CV folds. *AUROC* area under the ROC curve, *AUPRC* area under the precision-recall curve, *PPV* positive predictive value, *NPV* negative predictive value.

**Variable**	**CV-fold 1**	**CV-fold 2**	**CV-fold 3**	**CV-fold 4**	**CV-fold 5**	**Mean (SD)**
Best epoch	193	158	197	188	139	175.0 (±25.308)
Accuracy	0.883	0.870	0.866	0.861	0.887	0.873 (±0.011)
AUROC	0.931	0.936	0.946	0.923	0.943	0.936 (±0.009)
AUPRC	0.904	0.880	0.907	0.881	0.911	0.897 (±0.015)
Sensitivity	0.761	0.776	0.769	0.748	0.832	0.777 (±0.032)
Specificity	0.946	0.919	0.916	0.919	0.916	0.923 (±0.013)
PPV	0.881	0.834	0.828	0.829	0.838	0.842 (±0.022)
NPV	0.884	0.887	0.883	0.875	0.913	0.888 (±0.014)

3. Supplementary Figures

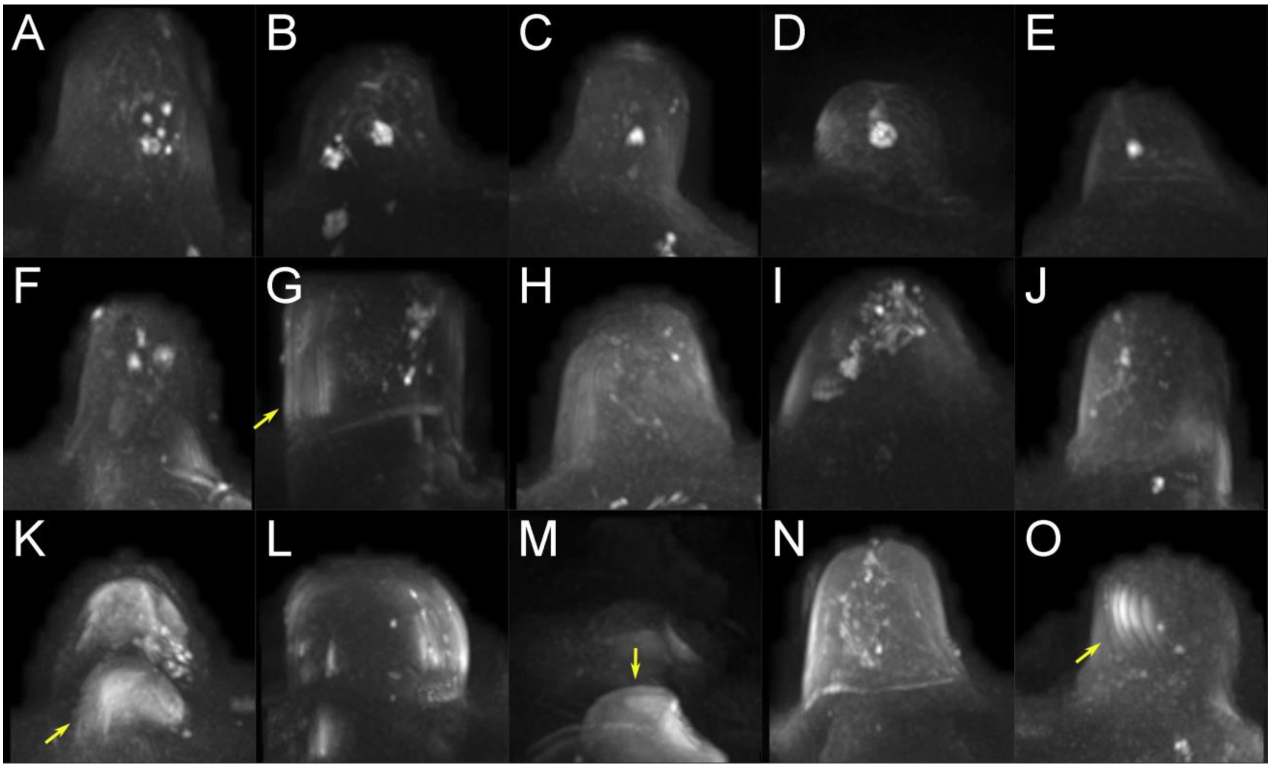

Figure S1: BI-RADS 5 lesions in clinical cases (examples). Each tile of the figure presents the left or right breast of one clinical case with a diagnosed BI-RADS 5 lesion. Row 1 (A-E) shows images without the presence of artifacts. Row 2 (F-J) shows images that contain artifacts with no or moderate influence on the diagnostic assessment. Row 3 (K-O) shows images with artifacts that significantly impede the diagnostic evaluation. Artifacts in DWI often originate from multiple technical and/or patient-related sources that may be interdepend and thus it is not always possible to attribute one specific artifact source. The arrows mark regions of artifacts within the images with possible contributing factors related to remaining surface coil flare (e.g. G, O), ghosting artifacts related to silicone implants (e.g. visible in K, M), and related to insufficient fat suppression (e.g. also visible in O).

4. References

1. Griswold MA, Jakob PM, Heidemann RM, et al. Generalized autocalibrating partially parallel acquisitions (GRAPPA). *Magn Reson Med.* 47(6):1202–1210. https://doi.org/10.1002/mrm.10171 (2002).
